# Photoactive Hybrid Catalysts Based on Natural and Synthetic Polymers: A Comparative Overview

**DOI:** 10.3390/molecules22050790

**Published:** 2017-05-12

**Authors:** Juan Carlos Colmenares, Ewelina Kuna

**Affiliations:** Institute of Physical Chemistry, Polish Academy of Sciences, Kasprzaka 44/52, 01-224 Warsaw, Poland; ekuna@ichf.edu.pl

**Keywords:** photoactive hybrid materials, photocatalyst, biopolymers, synthetic polymers, water/air detoxification, metal oxides

## Abstract

In the present review, we would like to draw the reader’s attention to the polymer-based hybrid materials used in photocatalytic processes for efficient degradation of organic pollutants in water. These inorganic–organic materials exhibit unique physicochemical properties due to the synergistic effect originating from the combination of individual elements, i.e., photosensitive metal oxides and polymeric supports. The possibility of merging the structural elements of hybrid materials allows for improving photocatalytic performance through (1) an increase in the light-harvesting ability; (2) a reduction in charge carrier recombination; and (3) prolongation of the photoelectron lifetime. Additionally, the great majority of polymer materials exhibit a high level of resistance against ultraviolet irradiation and improved corrosion resistance. Taking into account that the chemical and environmental stability of the hybrid catalyst depends, to a great extent, on the functional support, we highlight benefits and drawbacks of natural and synthetic polymer-based photocatalytic materials and pay special attention to the fact that the accessibility of synthetic polymeric materials derived from petroleum may be impeded due to decreasing amounts of crude oil. Thus, it is necessary to look for cheap and easily available raw materials like natural polymers that come from, for instance, lignocellulosic wastes or crustacean residues to meet the demand of the “plastic” market.

## 1. Introduction

**Figure 1:**
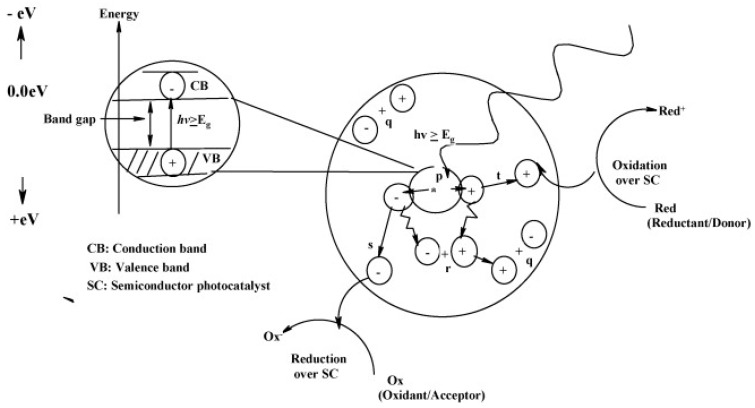
Photochemical and photophysical processes over photon-activated semiconductors, where: (p) is photogeneration of electron/hole pair, (q) is surface recombination, (r) is recombination in the bulk, (s) is diffusion of acceptor and reduction on the surface of semiconductor and (t) is oxidation is oxidation of donor on the surface of semiconductor particle. Reprinted from [31] with permission from Elsevier.

In recent years, the interest in photocatalysis as a green and eco-friendly method for pollution remediation [1,2], energy conversion [3,4] or chemical synthesis [5] has been increasing. However, the proficient application of photocatalysis is restricted to the fabrication of advanced nanostructured materials consisting of various types of semiconductors [6,7,8,9,10,11]. Current research in this field mainly concentrates on the development of hybrid materials containing photoactive metal oxides [9,10,11,12,13], quantum dots, or perovskites [14,15] to enhance the efficiency of photocatalytic systems [16]. In order to understand the main restrictions of photocatalytic performance, we have to know the main photochemical and photophysical mechanisms leading that process (Figure 1) [16,17,18,19,20]. It is worth mentioning that correlation between semiconductors and light plays a crucial role in the case of photocatalysis [12,16]. The absorption of photons with a specific energy allows for excitation of electrons from the valence band to the conduction band, producing hole–electron pairs responsible for redox processes. However, oxidation and reduction processes can be inhibited due to charges and radical recombination or back electron transfer processes [21,22]. Consequently, the employed photonic power is much higher than desirable rate of the desired chemical transformation, which is the main limitation of photocatalytic performance [23].

Significant features which can extensively improve photocatalytic efficiency and light absorption capability, are strongly correlated to size and structure of photocatalytic materials, their specific surface area, or crystalline phase [7,24]. Taking into consideration the fact that the required physicochemical characteristics are a consequence of various features of the main components and interaction between them [25,26], the enhancement of photocatalytic performances could be obtained through an organic–inorganic hybrid complex made of a semiconductor and suitable support [25]. Multiple material combinations provide synergistic effects that are able to create and improve properties of nanomaterials [27,28,29] which are beneficial for enhancing the efficiency of photocatalytic reactions. The synergistic effect originating from the combination of individual elements is clearly evident in the case of polymer-based hybrid photocatalysts. A great majority of polymer materials exhibits a high level of resistance against ultraviolet irradiation and improved corrosion resistance as well as environmental stability [30,31]. Compared with semiconductor oxides, the great majority of polymeric supports are chemically inert, mechanically stable, inexpensive and easily available. Additionally, the hydrophobic nature of polymer gives an advantage to congregate the organic pollutants on the surface and raise the efficiency of adsorption and subsequent degradation reaction rates [32]. Therefore, in recent years polymer-based hybrid materials have been emerging as promising device in the photocatalytic field.

Nowadays, plastic production is based mainly on feedstock derived from petroleum refineries. A wide range of synthetic or semisynthetic polymerization products are obtained from oil and gas, which undergo chemical processing [33]. As inputs for plastic manufacturing, refinery olefins (mainly propylene and less quantities of ethylene or butylenes) are produced from alkane transformations [34]. Unfortunately, there are difficulties with the discovery of new oil deposits, and some sedimentary basins that contain crude oil have already been explored. The oilfields which still have not been explored are located in inaccessible regions of the world [35]. Taking into consideration the fact that the plastic production is based mainly on feedstock derived from oil refinery, it is necessary to look for cheap and easily available raw materials like highly abundant biopolymers in nature. In this state-of-the-art review, we focus on synthetic and natural polymers, and highlight the main benefits and limitations coming from polymer materials which could be used as support for the fabrication of photocatalytic hybrid materials.

## 2. Synthetic and Natural Polymers

**Figure 2:**
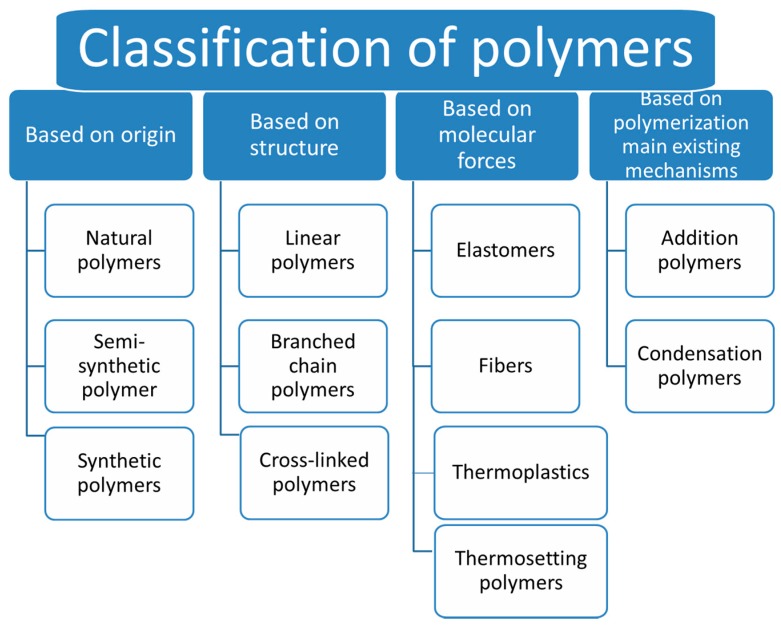
Classification of polymers according to [37].

According to IUPAC (International Union of Pure and Applied Chemistry) nomenclature, the word “polymer” refers to substances composed of macromolecules with high relative molecular mass. However, this term could be also be applied to polymer substances, polymer blends or polymer molecules [36]. Additionally, polymers include a wide class of materials which can be grouped according to source, functionalities, structure, thermal behavior, polymerization mechanism or preparation techniques (Figure 2) [37,38]. In this mini-review, we mainly focus on synthetic and natural polymers, which can find applications in photocatalysis, taking into consideration factors related to the photocatalytic properties, including stability, biodegradability, and biocompatibility with inorganic materials as well as following recent progress on the synthesis of hybrid materials.

**Figure 3:**
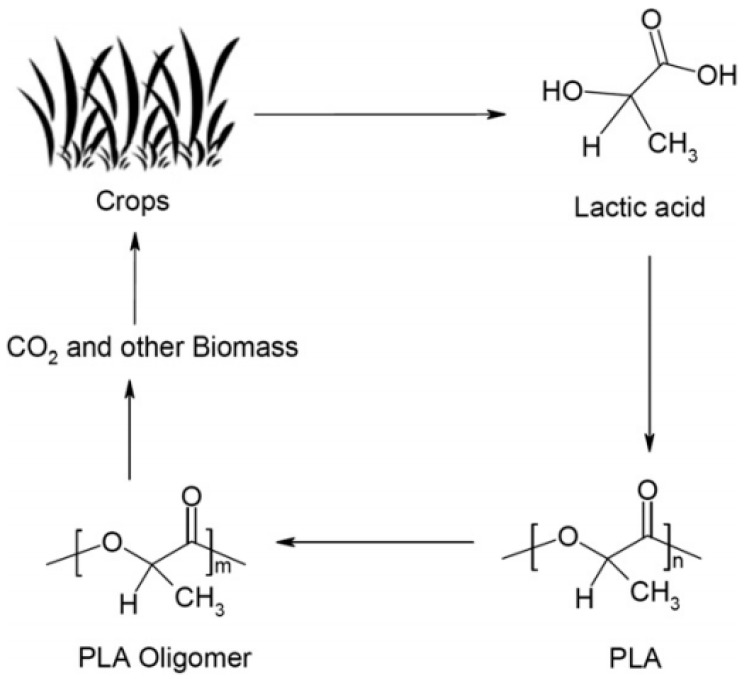
Life cycle of polylactide (PLA), an example of biodegradable synthetic polymers. Reprinted from [41] with permission from Elsevier.

Synthetic and semisynthetic polymers originating from crude oil have had a huge impact on modern science and technology due to their physicochemical properties. In many cases, chemical, physical and biological resistance play crucial roles in the selection of a desirable polymer for determined function. However, with respect to most synthetic materials, the affected time-resistant properties of polymeric wastes can lead to the release of toxic degradation products during decomposition which is not acceptable from an eco-friendly point of view [38]. Owing to concerns about the natural environment and shortages of non-renewable sources, the interest in polymers derived from natural sources like starch, lignocellulose or proteins is still increasing [34]. Biodegradable polymers that can be obtained from renewable resources (Figure 3) have emerged as environmentally friendly substitutes for non-biodegradable materials. It is worth mentioning that some of them exhibit similar or superior mechanical properties to petroleum-based polymers [39]. However, in many cases they possess inferior physical feature in terms of stability and strength and lots of them require high-cost production [33]. Additionally, in comparison with synthetic polymers, several natural polymers cannot be processed into a wide range of shapes, due to the fact that the high processing temperature destroys their structure [40]. Consequently, the design of new eco-friendly and highly efficient and stable photocatalytic biopolymer hybrid materials is challenging.

### 2.1. Photocatalytic Hybrid Materials Based on Synthetic Polymers

**Figure 4:**
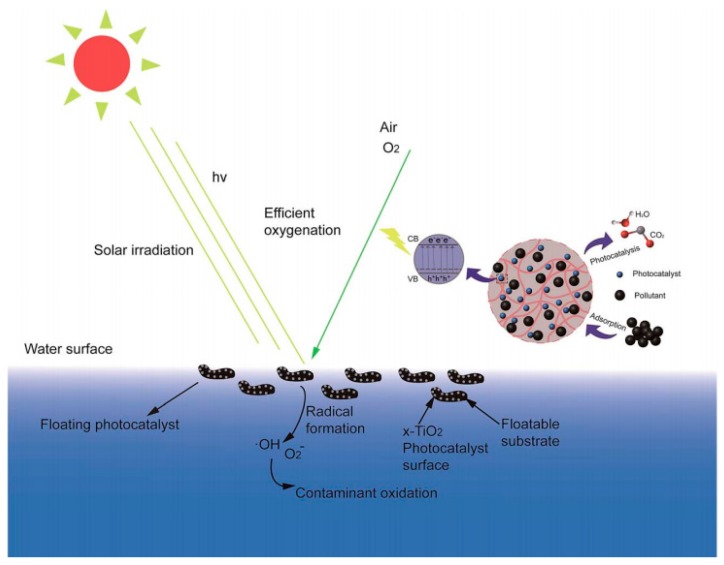
Schematic representation of a floating photocatalyst (CB: conduction band; VB: valence band). Reproduced from reference [70] by permission of the Royal Society of Chemistry.

Various types of synthetic polymer shave been reported as photocatalytic supports in the literature, namely: polyethylene (PE) [42], polypropylene (PP) [43], polystyrene (PS) [44], polyethylene terephthalate (PET) [45], polyvinyl chloride (PVC) [46], polyvinyl alcohol (PVA) [47], polycarbonate (PC) [48] and so on. To our best knowledge, the first attempt to produce polymer hybrid materials was made in 1995 by Tennakone [49]. Titanium oxide with polyethylene films as support was used for the photocatalytic decomposition of phenol with a high degradation ratio (50% after 2.5 h of illumination). Further, experimental studies on polypropylene non-woven with zinc oxide nanorods indicate that this kind of photocatalytic materials exhibited not only excellent catalytic activity but also high stability [50,51]. Hence, these hybrid materials can be successfully used for water treatment processes by acting as photocatalysts and filters at the same time [52]. Additionally, the synergetic effect of the combination of metal oxide and polymers allows for protection of the polypropylene fiber against surface cracks and limits the well-known photocorrosion process of zinc oxide [52,53]. Similar photoactive hybrid materials based on polybutylene terephthalate (PBT) polymer fiber mats were used for photocatalytic dye degradation. These studies confirmed that the catalyst supported on the polymer mat could be reused without a particular recovery step [54]. They also pointed out the fact that the combination of proper fabrication methods allows for better photocatalytic performance (Table 1, Entry 1) [55]. Another example of synthetic polymer hybrid materials, which have applications in water treatment processes, are polyethersulfone or polyvinylidene fluoride membranes with various types of metal oxides (e.g., titanium, zinc or chromium) displaying good antifouling performance, including photo-catalysis, self-cleaning, and filterability properties [55,56]. Our special attention gives merit to hybrid materials based on conjugated organic polymers (COPs) like polyaniline (PANI) [57] (Table 1, Entry 2), poly(pyrrole) (PPy) [58], polythiophene (PT) [59], polyacetylene (PA) [60], poly(methyl methacrylate) (PMMA) [61], polythiopene (PT) [62], polyparaphenylene (PPP) [63], polyparaphenylenevenylene (PPV), poly(3,4-ethylenedioxythiophene) (PEDOT) [63] or poly(*O*-phenylenediamine) (POPD)) [64]. The conjugated organic polymers are mostly p-type semiconductors, due to their electrical and optical properties. Specifically, their high electron mobility or high photon absorption coefficient under visible spectra has attracted increasing interest for photocatalytic applications, e.g., degradation of pollutants or hydrogen generation by water splitting [65]. In terms of water treatment processes, another interesting perspective solution offered by polymeric support is the possibility of fabricating a floatable photocatalyst, the concept of which is shown in Figure 4. These kinds of materials are able to maximize illumination utilization and oxygenation processes of the photocatalyst by approaching the air/water interface, which in the end can result in higher rates of radical formation and oxidation efficiencies [66].

Polymeric supports possess different morphologies and can exist in the form of sheets [67], nanospheres [68], or nanoparticles [69]. Of course in all these cases, polymer materials contribute to an increase the photocatalytic activity of inorganic–organic materials. However, contact surface area of the hybrid photocatalyst, which has a significant influence on their activity, is lower for fiber polymeric supports. Selected examples of catalysts based on polymeric fibers with high photocatalytic activity are shown in Table 1.

**Figure 5:**
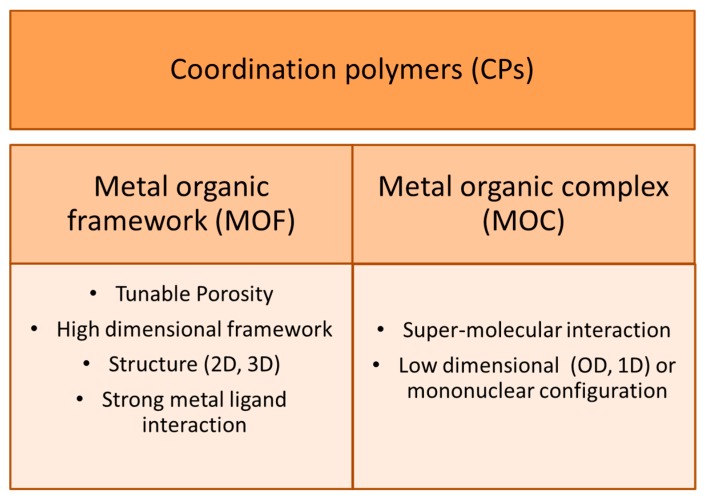
The coordination polymers which consist of the branch of metal organic framework and metal organic complex. Adapted and modified with permission from [72] Copyright (2012) American Chemical Society.

**Figure 6:**
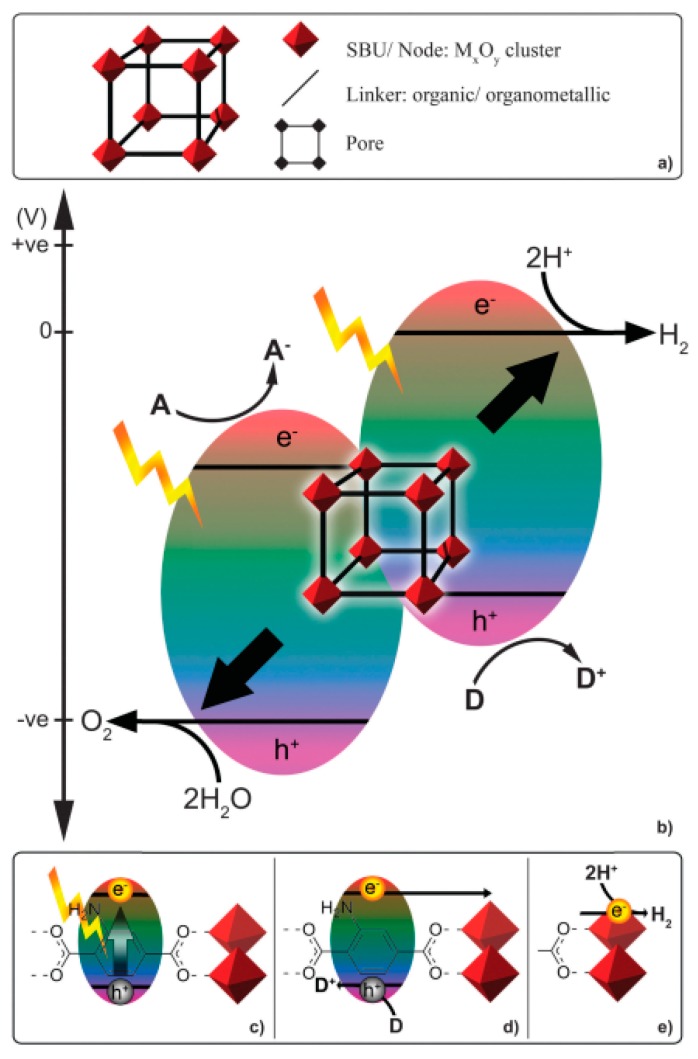
(**a**) the components of the MOF structure; (**b**) conceptual schematic for photo-catalyzed water oxidation or reduction using a MOF in the presence of an acceptor or donor; (**c**) light harvesting accomplished by an organic linker; (**d**) generation of a charge separated state and quenching of h^+^ by a donor; (**e**) electron transfer to the metal oxide node and subsequent proton reduction (SBU: secondary building units). Reproduced from reference [73] with permission of The Royal Society of Chemistry.

It is worth mentioning that in the open literature a new class of hybrid materials, represented by coordination polymers (CPs) and composed of metal clusters with organic ligands (Figure 5), can be used in photocatalysis. These crystalline materials possess high dispersion of active sites, tuneable adsorption properties, appropriate pore size and topology [71]. Additionally, the effective solar light absorption properties can be obtained through modifying the composition of the metal cations and organic linkers. The possibility to connect the light-harvesting and catalytic components allows for conversion of solar energy to chemical energy by artificial photosynthesis [72]. Materials based on coordination polymers provide crucial information about synergistic effects derived from multiple elements of hybrid materials and allow for understanding the fundamental principles about light harvesting and energy transfer phenomena, schematically explained in Figure 6. In spite of some examples of CP-based heterogeneous photocatalytic systems [73], until now the CP-based soluble complexes have mainly been used in homogenous catalytic processes which is a serious limitation for broad industrial application due mostly to the problem of photocatalyst filtration after the process.

### 2.2. Photocatalytic Hybrid Materials Based on Natural Polymers

**Figure 7:**
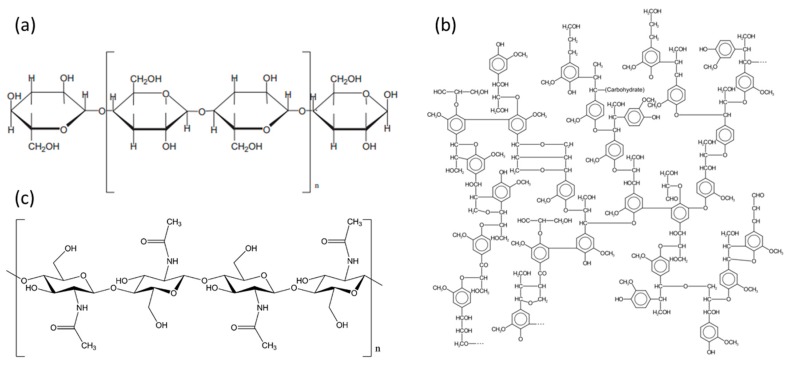
Structure of most common natural polymers: cellulose (**a**), lignin (**b**) and chitin (**c**). Reprinted from references [83] with permission from Elsevier.

**Figure 8:**
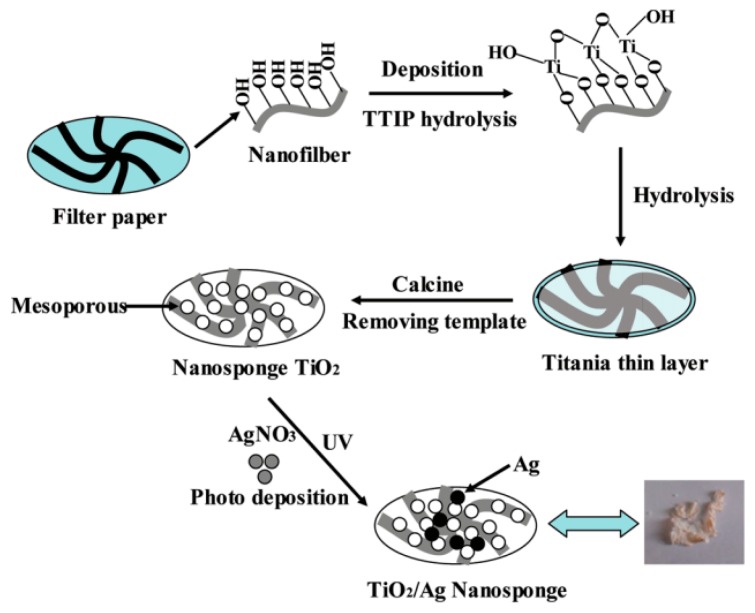
Schematic illustration of the synthetic approach for TiO_2_/Ag nanosponge materials (TTIP: titanium *iso*-propoxide). Reprinted with permission from [86]. Copyright (2012) American Chemical.

Natural polymers derived from renewable resources or waste products can also serve as a desirable organic support for inorganic semiconductor metal oxides [77,78,79]. Polysaccharides, lignin, cellulose, hemicellulose, chitin, chitosan, starch or xylan, possess excellent sustainability, biodegradability and can be used as abundant industrial raw feed stock to synthesize photocatalytic hybrid materials [80,81,82]. Natural polymers (see structures in Figure 7) are mainly obtained by extractions from wood, plants or even residues of living organisms [83], that make them more attractive in comparison with synthetic polymers. Natural fibers are frequently used as a reinforcing composite for producing hybrid materials because they exhibit advantages, like recyclability and eco-friendliness, over their synthetic counterparts [84]. Additionally, natural fibers possess a higher volume fraction, and thus a larger loading capacity [85]. For these reasons they are widely used to produce composite materials, especially in the field of photocatalysis. For instance, depositing titanium dioxide on cellulose fiber surface allows for obtaining hybrid materials with a high degradation ratio of organic compounds like organic dye or phenolic contaminants [86] (Table 2, Entry 1). Yu et al. obtained cellulose-templated TiO_2_/Ag nanosponge composites with enhanced photocatalytic activities for the degradation of RhB; the synthetic procedures for this material are shown in Figure 8 [86]. The polymeric nanocomposite membranes with cellulose fibers can be also used for gas separation processes (e.g., hydrogen recovering, nitrogen generation or carbon dioxide separation) [87,88]. However, due to the fact that cellulose consists of monosaccharide units (Figure 7a), it is hydrophilic and exhibits a rather poor interaction with most of the non-polar compound. Many efforts have been done to obtain uniform dispersion of the fibers within the matrices. Furthermore, it is worth noting that plant fibers like cellulose possess relatively low processing temperatures (<200 °C), and for this reason, researchers have used low-temperature techniques (e.g., sol-gel method, hydrothermal method, dip coating method, etc.) to coat natural fibers [89]. Despite this, in the open literature we can find successfully completed examples of many attempts to modify surface properties of natural fibers (see Table 2).

In contrast to hemicellulose and cellulose, lignin possesses rather a complex structure due to the different types of linkages connecting the phenylpropanoid-based units [90] (Figure 7b) and is seen as a product with little intrinsic value but potentially, for instance, can serve as a stable biopolymeric support for hybrid materials [91]. It should be noted that lignin can be used for the synthesis of functional hybrid materials with antimicrobial properties [92] as well as adsorptive properties for the removal of inorganic compounds from aqueous solutions [93]. However, the stability of biopolymers based on lignin under photocatalytic conditions is not high. Some critical review reports pointed out that photocatalytic methods can be used also to degradate lignin and lignin-based phenolic compounds [94]. From this point of view, it is interesting to study if lignin, which consists of phenylpropanoid-based structures, can be used as a support for the fabrication of stable hybrid photocatalysts with the aim of being applied for mineralization of organic contaminants in water.

Chitin (Figure 7c) represents the next most plentiful natural polysaccharide and is source of chitosan which is obtained by partial deacetylation of chitin. Due to the presence of various functional groups, chitosan can be applied as an adsorbent for the removal of different kinds of pollutants [95,96]. Additionally, this polymer can be used in the wide range of form, e.g., hydrogels [97], nanofiber mats [98], and nano-beads as well as powders [99]. Chitosan may have a high specific surface area as a bead or fiber, and thus exhibits strong adsorption capacities. However, all of these forms depend on the fabrication method. The formation of a highly specific surface area requires mainly specialized methods like electrospinning [100]. In the open literature, one can find the description of many other facile methods to obtain hybrid catalysts like those based on titanium dioxide [100], zinc oxide [101] (Table 2), and niobium oxide [102] among others, combined with chitosan, which can be used for water treatment processes. However, chitosan, as well as other polysaccharides have some limitations associated with wastewater treatment application, namely: they possess high swelling capacities and low resistance, especially in extreme wastewater conditions (e.g., acid medium), which may result in significant leaching. Consequently, the catalyst based on these natural polymers cannot be stable and may promote the expansion of organic matter in wastewater [103].

## 3. Summary and Future Perspectives

The selected studies in this short overview serve as clear examples that both natural and synthetic polymers can be successfully used in the field of hybrid innovative materials for heterogeneous photocatalysis in the context of pollutant degradation. The organic–inorganic hybrid materials exhibit significantly better photocatalytic properties than the separate components, due to the synergistic effect coming from the intrinsic properties of a photoactive semiconductor and polymers. Several key advantages can be expected from polymeric support, such as: (a) an increase of the specific surface area which consequently allows for adsorption of higher amounts of target pollutants [109,110,111], and (b) an improvement of the photocatalytic performance by promoting reduction of the charge carriers recombination and prolongation of the photoelectron lifetime [112]. In this mini-review, the highlighted benefits and drawbacks of natural and synthetic polymer-based photocatalytic materials will exponentially increase in importance due to the fact that accessibility of synthetic polymeric materials derived from oil, gas and carbon (non-renewable sources) will be impeded because of the decreasing amount of fossil resources. Thus, this fact turns on the alarm to look for cheap and easily available raw materials like bio-polymers that come from sources such as lignocellulosic wastes or crustacean residues to cover the increasing demand of the market for plastics. Currently, the scientific world indicates that polymer materials are the key promising components of the next generation of photocatalytic hybrid materials for water and air treatment processes [113,114]. However, there are still some limitations on this topic (Table 3) that should be studied extensively.

## Figures and Tables

**Table 1:** Selected photocatalytic hybrid materials based on synthetic polymers used for degradation of organic contaminants.

Entry	Polymer Hybrid Materials	Target Contaminant	Light Source	Fabrication Method	Photocatalytic Behavior	Ref.
1	ZnO nanorods on polybutylene terephthalate (PBT) polymer fiber mats	Azo organic dye (acid red 40)	Ultraviolet (UV) radiation in the range of 320–390 nm providing 79 mW/cm^2^ of energy flux.	Thin films formed by low temperature vapor phase atomic layer deposition (ALD) and hydrothermal growth of ZnO nanorod crystals on a seed layer.	Degradation ratio ~90% of the dye within 2 h. The combination of ALD and hydrothermal method allow to obtain the best performance of the photocatalyst and may be also used for other crystal growth systems, such as TiO_2_, Fe_2_O_3_, SnO_2_ and V_2_O_5_, where high area and ready solution access are desired.	[55]
2	ZnO nanoparticles on wool and polyacrylonitrile (PANI) fibers	Methylene blue (MB) and eosin yellowish (EY) dye	High-pressure mercury lamp covers illumination spectrum ranging from ultraviolet to visible (200–800 nm).	Impregnation of polymeric fibers using sol-gel process at ambient temperature. ZnO-sol is based on the method described in the literature with minor changes in details.	There is 77% MB dye degradation after 6 h upon ZnO/PANI and 80% upon ZnO/wool fibers, which is 4-fold more in comparison to bare fibers. Similar results of degradation were obtained for EY dye, where the degradation ratios equal 64% and 50%, respectively.	[57]
3	CeO_2_-ZnO-polyvinylpyrrolidone (PVP)	Rhodamine B (RhB)	UV lamp (8 W) with emission wavelengths at 254 nm.	The electrospinning technique was followed by thermal treatment to obtain CeO_2_–ZnO nanofibers. The nonwoven mat was prepared from the precursor solution of PVP/Ce(NO_3_)_3_/Zn(CH_3_COO)_2_.	After 3 h of irradiation, only 17.4% and 82.3% of Rhodamine B was decomposed catalyzed by pure CeO_2_ and ZnO fibers, respectively, whereas almost 98% was decomposed applying the CeO_2_–ZnO-composite fibers.	[74]
4	ZnO nanowires on polyethylene (PP)	Methylene blue (MB)	UV light source (6 W)	ZnO nanowires were grown from seed ZnO nanoparticles affixed onto the commercially available fibers by hydrothermal method.	After 2.5 h of irradiation, ZnO/polyethylene fibers degraded 83% of the MB, whereas the fibers without ZnO degradate only 32%. 24% of MB was found undergo self-degradation under the same UV light without using polyethylene fibers.	[75]
5	ZnO/SnO_2_-polyvinylpyrrolidone (PVP)	Rhodamine B	High-pressure mercury lamp (50 W) with main emission wavelength at 313 nm.	A simple combination method of sol-gel process and electrospinning technique. The electrospun composite nanofibers was obtained by the precursor solution of PVP/ZnCl_2_/SnCl_2_.	After 50 min, the degradation efficiency of RhB was equal to 75, 35, and 85% for ZnO, SnO_2_, and TiO_2_ fibers, respectively. However, the time for complete decolorization of dye solution over the ZnO/SnO_2_-nanofibers was 30 min.	[76]
6	Reduced graphene oxide/titanium dioxide filter (RGO/TiO_2_) and reduced graphene oxide/zinc oxide filter (RGO/ZnO) on polypropylene(PP) porous filter	Methylene blue (MB)	Halogen lamp (150 W)	The polypropylene (PP) porous filter was incorporated with reduced graphene oxide (RGO) and metal oxides via a simple hydrothermal approach.	The combination of RGO and the metal oxide compounds on the filters shows more than 70% of MB adsorption in 20 min compared with those consisting of individual materials, degradation after 120 min 99%.	[50]

**Table 2:** Selected photocatalytic hybrid materials based on natural polymers used for degradation of organic contaminants.

Entry	Polymer Hybrid Materials	Target Contaminant	Light Source	Fabrication Method	Photocatalytic Behavior	Ref.
1	Titanium dioxide (TiO_2_) immobilized in cellulose matrix	Phenol	UV (6 W) light at wavelength of 254 nm was used. The mean light intensity equal to 0.56 mW/cm^2^.	Composite films have been prepared via a sol-gel method.	The composite films exhibited high degradation ratio (90% after 2 h of irradiation) without remarkable loss of photocatalytic activity after three times.	[85]
2	ZnAc/cellulose acetate (CA) composite nanofibers	Rhodamine B and phenol	Ultraviolet lamps (PHILIPS 365 nm) as the irradiation source.	Electrospinning technique in combination with calcination.	Almost 100% of Rhodamine B and 85% phenol (after 24 h) was decomposed in the presence of TiO_2_/ZnO composite nanofibers under mild conditions.	[104]
3	ZnO/cellulose hybrid nanofibers	Methylene blue (MB) and eosin yellowish (EY) dye	Tungsten lamp (500 W) was used as the visible light source.	A novel method that combines electrospinning and solvothermal techniques	Nearly 50% of Rhodamine B was decomposed after 24 h of irradiation under visible light.	[105]
4	Photoactive TiO_2_ films on cellulose fibers	Methylene blue (MB) and heptane-extracted bitumen fraction (BF) containing a mixture of heavy aromatic hydrocarbons	Reproducible solar light (50 mW/cm^2^).	Sol-gel method	The degradation ratio of MB reached 90% after 20 h and 90% for BF fraction after 9 h without loss of activity after three illumination cycles.	[106]
5	Rice-straw-derived hybrid TiO_2_–SiO_2_ structures	Methylene blue (MB)	UV-A (8 W) lamps (300–450 nm) providing an irradiation power flux of 2.0 mW/cm^2^.	Impregnation method.	The photocatalytic decomposition of methylene blue after 90 min obtained was 100%.	[107]
6	Chitosan (CS)-encapsulated TiO_2_ nanohybrid	Methylene blue (MB)	UV light at a wavelength of 365 nm.	Nanohybrid materials was prepared by chemical precipitation method.	The catalyst showed high photocatalytic activity of 90% degradation after 3 h of irradiation and without losing photocatalytic activity after five recycle tests.	[100]
7	Fe_3_O_4_/chitosan/TiO_2_ nanocomposites	Methylene blue (MB)	Illumination with UV light.	Facile and low-cost method by solvents thermal reduction.	The degradation rate of methyl blue was 93% after 30 min for Fe_3_O_4_/CTS/TiO_2_ nanocomposites.	[108]

**Table 3:** The main pros/cons of using synthetic polymers and biopolymers for photocatalytic hybrid materials.

	Synthetic Polymers	Biopolymers
**Availability**	Decreasing	High
**Physicochemical resistance**	High	Low
**Thermal stability**	High	Low
**Large-scale applications**	Possible	Difficult
**Environmental-friendly**	No	Yes
**Cost of production**	Low	High
**Sustainability**	Low	High
